# External Ventricular Drain Column Height Fails to Explain Cerebrospinal Fluid Drainage Volumes

**DOI:** 10.1111/nicc.70189

**Published:** 2025-10-11

**Authors:** Yuan Li, Shripal Gunna, Amber Salter, Yohan Kim, DaiWai M. Olson

**Affiliations:** ^1^ Affiliated Hospital of Zunyi Medical University Zunyi China; ^2^ University of Texas Southwestern Dallas Texas USA

## Abstract

**Background:**

An external ventricular drain (EVD) facilitates cerebrospinal fluid (CSF) diversion and intracranial pressure (ICP) monitoring. CSF drainage can be regulated by controlling the fluid drain height. There is limited evidence guiding nursing practice for adjusting drain height when caring for patients with an EVD.

**Aim:**

This purpose of this study is to explore the relationship between EVD drain height setting and CSF drainage volumes.

**Study Design:**

This was a retrospective observational study. The initial 14 days from EVD placement for patients admitted to a university hospital between 2022 and 2024 who received an EVD and had CSF diversion were considered. Associations between time‐stamped data for five drain height categories (< 0 cmH_2_O, equal to 0 cmH_2_O, 5 cmH_2_O, 10 cmH_2_O and 15 cmH_2_O) and mean daily CSF drainage were analysed using a linear mixed model with Turkey adjustment.

**Results:**

The 115 patients provided 27 552 time‐stamped observations over a median of 6.3 (IQR = 2.7–11.8) EVD days. The mean age was 51 (15.5) years; 53.0% were female, 64.9% were White and 74.8% were Non‐Hispanic. Across the first 14 EVD days, the mean daily CSF volume was 179.2 (121 mL) with a range from 0 to 539 mL. Mean daily CSF output by height were: < 0 cmH_2_O (180.0 [138.8]), 0 cmH_2_O (164.1 [133.7]), 5 cmH_2_O (162.0 [105.5]), 10 cmH_2_O (194.7 [116.2]) and 15 cmH_2_O (174.9 [123.4]). The mixed model showed no association between mean daily CSF output and drain height category (*p* = 0.443).

**Conclusion:**

This study found a limited role of low‐altitude settings in facilitating CSF drainage in EVD management.

**Relevance to Clinical Practice:**

Future efforts should be made to establish if there is a relationship between ICP management based on CSF drainage volume or drain height setting and to examine the impact of CSF drainage on patient outcomes. This information may also guide clinicians in making decisions about CSF drainage.

**Impact Statements**

- What is known about the topic
  ○ EVDs are commonly placed in patients with increased ICP to serve as a CSF pressure flow diverter.
  ○ The drain height of EVD is hypothesized to impact the amount of CSF drainage.
  ○ There is a lack of information on the association between EVD height setting and CSF drainage volume.
- What this paper adds
  ○ Drainage height does not explain the changes in CSF drainage volumes, which pose a fundamental challenge to the traditional clinical practice of regulating CSF drainage volume based on a fixed height gradient.
  ○ EVD management should consider multiple factors, which may play a more important role in regulating CSF drainage volume.
  ○ On the basis of maintaining the basic height gradient, a multi‐parameter driven approach may achieve a more precise drainage.

## 1 Introduction

An external ventricular drain (EVD) is used to monitor intracranial pressure (ICP) or divert cerebrospinal fluid (CSF) in acute hydrocephalus, intraventricular haemorrhage (IVH), aneurysmal subarachnoid haemorrhage (SAH), cerebral oedema, ventriculitis and severe traumatic brain injury (TBI) [1]. Acute brain injury is a leading cause of morbidity and mortality worldwide and a major public healthcare burden in the United States [2, 3]. Aneurysmal SAH remains a devastating condition, with a reported mortality rate up to 36% and an elevated chance of poor long‐term functional outcomes in survivors [4]. TBI and SAH commonly present with hydrocephalus due to obstruction of CSF passage across the ventricular system in the brain [2, 5]. Elevated ICP due to disrupted CSF dynamics is a critical concern for secondary brain injury, and EVDs are commonly placed in patients with increased ICP to serve as a CSF pressure flow diverter [6, 7, 8]. The practice of CSF diversion to reduce ICP and facilitate cerebral blood perfusion is often considered a life‐saving treatment in patients with elevated intracranial hypertension [9].

There is limited agreement on CSF drainage with regard to frequency, setting, duration and cessation [10, 11, 12]. Many providers advocate for a multidisciplinary approach where the neurosurgeon prescribes a height (typically referenced to an external landmark) that nurses use to adjust the drainage port such that the drain height helps to regulate CSF drainage and ultimately ICP [9, 13, 14]. Depending on the manufacturer, this is achieved by raising or lowering the drainage chamber of the EVD system and alternating between keeping the drain open or closed [15, 16, 17]. The drain height of EVD is believed to affect the amount of CSF drainage [18]. However, studies have shown inconsistent results on the relationship between drainage height and CSF in the current literature [19]. EVD management practice among institutions and practitioners varies greatly, with little evidence supporting ideal ICP recording and CSF drainage practices [6].

Experiments and analytical models show that the error in pressure measurement increases linearly with flow rate; however, this error is not clinically significant, regardless of drip chamber height [15, 20]. There is a lack of information on the association between EVD height setting and CSF drainage volume.

## 2 Aim of the Study

The purpose of this study is to explore whether the drain height setting of an EVD is associated with CSF drainage volumes. The primary hypothesis is that lower drain height is associated with increased CSF drainage rates.

## 3 Design and Methods

### 3.1 Design

This is a retrospective observational study. The Strengthening the Reporting of Observational Studies in Epidemiology (STROBE) guideline was used to report the study. The study is approved by the university institutional review board as exempt from consent because deidentified data are extracted after patient discharge. The data included in this study were collected as part of an ongoing registry (ENDPANIC, with ClinicalTrials.gov (NCT02804438#)) and the data collection methods have been previously described [21].

### 3.2 Population

For this analysis, subject data were included for all patients admitted to the Neuroscience Intensive Care Unit (NSICU) from January 2022 through December 2024 who received an EVD and had CSF diversion as part of their treatment. Subjects were included if they were 18 years of age or older and admitted to the NSICU for treatment of aSAH. Subjects and their data were included in the analysis if they were admitted to the NSICU with a diagnosis of aneurysmal subarachnoid haemorrhage and had an EVD placed for the purpose of CSF diversion as part of their medical treatment. Persons who were < 18 years of age were excluded from the analysis.

### 3.3 Settings

The practice at our institution is that the EVD drain column height—measured as the height (in cm) above the tragus—is determined by a neurosurgeon and set by nursing [22]. The height is referenced to the tragus of the ear as an external landmark that approximates the foramen of Monro. The nurses will leave the drain open continuously except during intra‐hospital transport and when measuring the patient's ICP (typically hourly) [23].

### 3.4 Data Collection

Demographic information, primary diagnoses, important laboratory tests, patient outcomes, clinical severity scores (Apache II, SOFA) and sedative medications were recorded. Only the first 14 days of EVD use after placement were included. Data for subjects meeting inclusion were extracted from the primary registry and uploaded to SAS v9.4 for Windows (SAS Institute) and all data analyses were completed using SAS. The observation date and time information included year, month, day, hour, minute and second (e.g., 09AUG21:10:27:41) based on EMR documentation time. Hourly and daily CSF volumes were calculated based on the number of minutes EVD placement (e.g., 0–60 min from placement was hour 1, and 0–1440 min from placement was EVD day 1). Five height categories were included: drain height set below 0 cmH_2_O, equal to 0 cmH_2_O (reference category), 5 cmH_2_O, 10 cmH_2_O and 15 cmH_2_O. Any observations with the EVD drain height set at or above 20 cmH_2_O were considered clamping trials and excluded from the analysis.

### 3.5 Data Analysis

Data for each variable were first examined for frequency (percent), mean (standard deviation) or median (interquartile range) as appropriate. The association of EVD height with increased CSF drainage rates was evaluated using generalised linear mixed models controlling for the repeated observations within subjects, an autoregressive (1) covariance structure and the interaction between day (1–14) and drain height as a random effect. Pairwise comparisons between drain heights used the Turkey's Honestly Significant Difference (HSD) test to control for multiple comparisons.

## 4 Results

The 115 patients provided 27 552 time‐stamped observations. Of these observations, 22 662 were within the 14 days after EVD placement and 3276 were excluded with drain heights at 20 cmH_2_0. The 19 386 observations included represent 923 days of monitoring with a median of 5.0 (2.8–9.0) days per patient. As shown in Table 1, the mean age was 51 (15.5) years; 61 (53.0%) were female, 74 (64.9%) were White, 17 (14.9%) were Black and 86 (74.8%) identified as non‐Hispanic. The most frequent reason for EVD placement was haemorrhagic stroke (58 [48.7%]). At the time of hospital discharge, 65 (56.5%) patients had an mRS ≥ 3. The mean NSICU length of stay, 16.4 (20.6) days, was not normally distributed; the median NSICU stay was 12 (4–21) days.

**TABLE 1:** Patient demographics.

Characteristic		Value^a^
Mean intensive care length of stay (days)		16.4 [20.6] (range 0.1–186)
Mean age (years)		51 [15.5]
Gender	Female Male Other or not given	61 (53.0%) 53 (46.1%) 1 (0.9%)
Race	Black White Asian Native American Pacific Islander Other or not given	17 (14.9%) 74 (64.9%) 5 (4.4%) 1 (0.9%) 5 (4.4%) 13 (11.5%)
Ethnicity	Hispanic Non‐Hispanic Other or not given	19 (16.5%) 86 (74.8%) 10 (8.7%)
Diagnosis category	Haemorrhagic stroke Ischaemic stroke Mass effect lesion	56 (48.7%) 9 (7.8%) 50 (43.5%)
Discharge modified Rankin scale score	0 1 2 3 4 5 6	18 (15.8%) 24 (21.1%) 7 (6.1%) 12 (10.5%) 16 (14.0%) 20 (17.5%) 17 (14.9%)

^a^ Reported as mean [SD] or frequency (percent).

**FIGURE 1:**
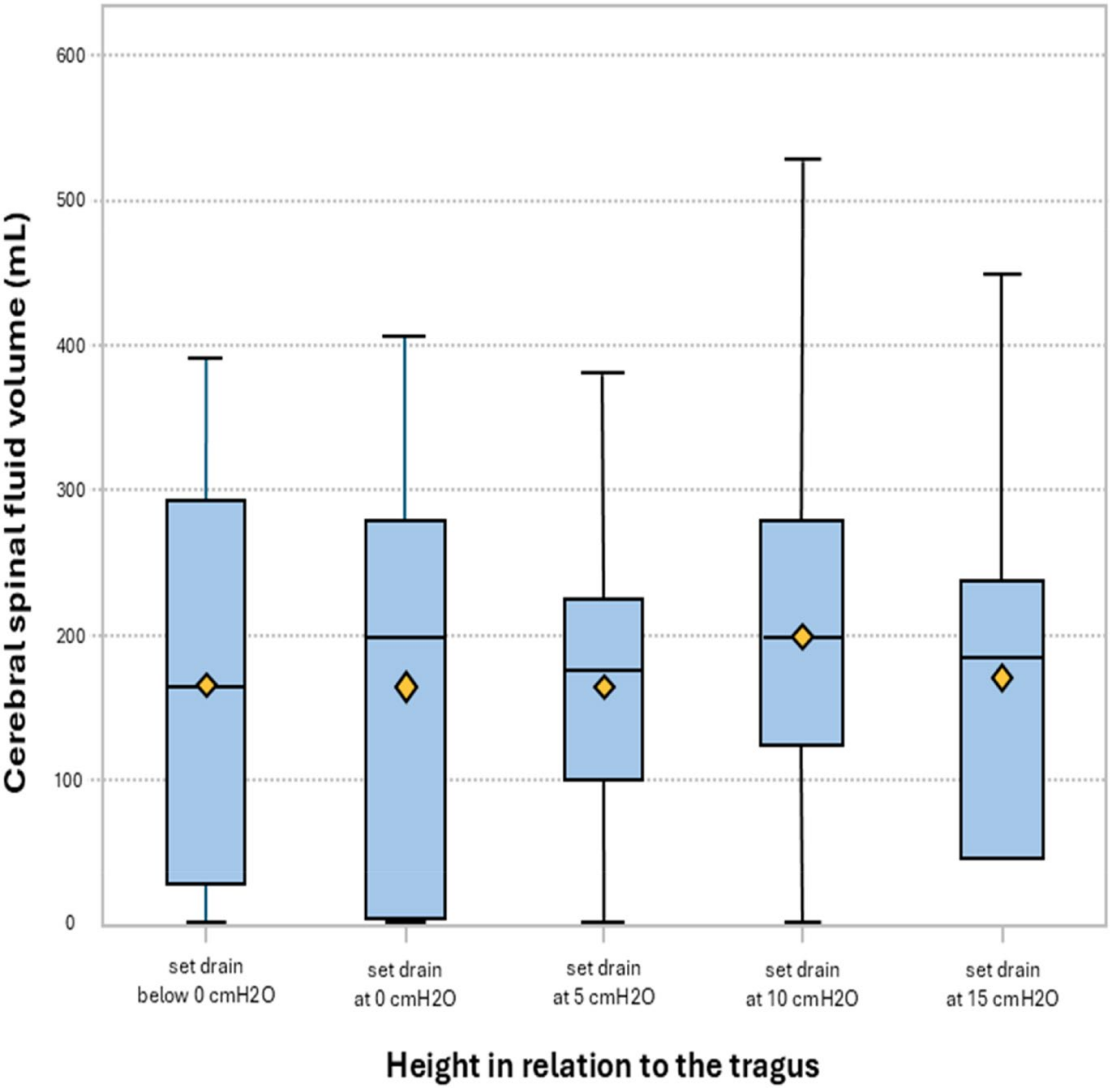
Box plot showing daily cerebrospinal fluid output volumes at each drainage height.

The mean daily CSF volumes over the first 14 days was 179.2 (121; range 0–539). The estimated mean daily CSF output volumes at each height are shown in Table 2. In an omnibus test controlling for repeated observations, there was no statistically significant difference in mean daily CSF volumes at different drain heights among the 923 observations during the first 14 days (*p* = 0.503). Controlling for age, race, diagnosis or NIHSS score did not improve model fitting. Figure 1 and Table 3 provide results from the mixed regression model with Turkey's HSD demonstrating that pairwise comparison of drain height does not explain differences in mean daily CSF output.

**TABLE 2:** Estimated mean daily cerebrospinal fluid output volumes at each drain height setting.

Drain height setting	Estimate (SE)	*Mixed regression linear model
Drain set at 15 cmH_2_O	143.6 (12.92)	< 0.0001
Drain set at 10 cmH_2_O	151.3 (11.07)	< 0.0001
Drain set at 5 cmH_2_O	139.8 (16.85)	< 0.0001
Drain set at 0 cmH_2_O	172.0 (35.12)	< 0.0001
Drain set below 0 cmH_2_O	132.0 (13.61)	< 0.0001

^*^ Controlling for repeated measures.

**TABLE 3:** Mixed regression linear modelling for multiple comparisons at different drain height settings.

Height 2 (cmH_2_O)	Height 1 (cmH_2_O)	Estimate	Standard error	*p*
Below 0	0	40.022	34.055	0.963
5	Below 0	−32.247	37.567	0.911
5	0	7.775	17.860	0.992
10	Below 0	−20.733	35.627	0.977
10	0	19.288	13.591	0.618
10	5	11.513	16.222	0.953
10	15	7.654	11.520	0.963
15	Below 0	−28.388	36.433	0.936
15	0	11.634	15.755	0.947
15	5	3.859	18.333	0.999

*Note:* Turkey adjusted *p*‐value.

## 5 Discussion

The data reveal a growing paradox in EVD management; drain height settings appear to have little impact on CSF drainage. Cerebral blood flow and ICP are dynamic values, and it is important to understand that the biomechanics of height‐dependent drainage are based on data from a static models [24]. This study demonstrates that during the first 14 days of EVD management, although the average daily drainage volume was 179.2 (121) mL, the daily CSF output was relatively stable across all drain heights. Moreover, the drain height setting of 10 cmH_2_O was found to have higher CSF daily mean volumes than lower height settings (Table 3 and Figure 1). This phenomenon may be due to the influencing mechanism of three dimensions. First, dynamic ICP fluctuations may form a driving force field in the opposite direction to the hydrostatic pressure difference [25]. When ICP rises sharply, even under high‐level drainage settings, the actual effective pressure gradient may still drive excessive drainage [20]. Second, CSF is generally produced relatively slowly (0.35 mL/min or 500 mL/day), and specific conditions alter an individual's CSF secretion rate from the choroid plexus CSF [24, 26]. The CSF drainage volumes may be dependent on available intracranial CSF. Third, changes in the compliance of the ventricular system may form nonlinear drainage resistance, especially in patients with haemorrhagic stroke (accounting for 48.7% of this cohort). These pathological changes, such as blood clot blockage and brain tissue displacement, can significantly change the fluid dynamics characteristics of the drainage system [27, 28].

The current practice of adjusting height to control flow is largely driven by the Monro‐Kellie hypothesis (doctrine); a hypothesis that is increasingly being identified as inadequate to explain cerebral blood flow, ICP and CSF dynamics [29, 30]. The height‐flow decoupling phenomenon observed in our study may be due to several clinical realities. First, clinicians often dynamically adjust the height setting according to the instantaneous ICP value, forming a treatment bias (confounding by indication) [4, 9]. Second, the main indication for EVD in half of the patients in this cohort was haemorrhagic stroke. Such patients often had acute hydrocephalus, and their CSF drainage needs essentially reflected the degree of mechanical obstruction of the cerebrospinal fluid circulation pathway under pathological conditions [31], rather than simply being driven by pressure gradients. It is worth noting that the median ICU stay was 12 days (IQR 4–21), suggesting that most patients experienced a pathophysiological transition from the acute phase to the subacute phase [4], and the time‐varying characteristics of CSF dynamic parameters during this process may mask the independent effect of the height setting.

This study found that 56.9% of patients had mRS ≥ 3 at discharge, highlighting the importance of optimising EVD management for improving prognosis. Based on the results, we propose exploring a dynamic pressure‐volume integrated management approach. On the basis of maintaining the basic height gradient (usually recommended 10–15 cm above the tragus level), continuous ICP monitoring, brain tissue oxygenation monitoring, cerebral blood flow monitoring and CSF drainage curve characteristics should be combined for real‐time adjustment [19, 32, 33]. Especially in patients with haemorrhagic stroke, personalised management protocols could be initiated when the daily CSF drainage volume is > 300 mL for 24 h—indicating a possible risk of over‐drainage [9]; or pathological B‐waves are present in the ICP waveform—indicating limited intracranial compliance [34, 35, 36]. A multi‐parameter driven approach such as this may achieve more precise drainage than relying solely on height adjustment. Further future prospective studies or RCTs that may need to be performed to contextualise these findings in clinical practice.

### 5.1 Limitations of the Study

The primary limitation to this analysis is the retrospective nature of the data obtained from chart extraction. However, these data do represent real‐world data, and automated data extraction allowed for a more accurate time‐stamp. Some clinical events that were not measured, such as craniectomy, infection and vasospasm, could have influenced CSF drainage. It is difficult to completely eliminate confounding in this design; factors such as CSF viscosity, the timing of nursing interventions or equipment‐related dysfunction (e.g., EVD blockage) [37, 38, 39]. The use of a heterogeneous sample is both a limitation and a benefit. The mixed regression model allowed us to control for repeated measurement and patient heterogeneity but does not rule out that drain‐height adjustment is helpful for specific individuals or diagnosis subsets.

## 6 Conclusion and Practice Implications

The EVD drain height setting does not appear to regulate or predict CSF drainage volumes in a heterogeneous sample of critically ill patients requiring external CSF diversion. This finding challenges the paradigm of traditional neurocritical care that relies on a fixed height gradient to regulate drainage volume. The findings further support the need to establish a more robust theoretical basis for the precision practice of neurocritical care. EVD management should consider multiple factors, such as the patient's pathophysiological status, EVD placement and nursing interventions, which may play a more important role in regulating CSF drainage volume. Designing a new management framework based on multimodal neurological monitoring with time‐varying adjustments individualised based on risk assessment would be a positive paradigm shift from the ‘empirical height regulation’ to a ‘pathophysiologically driven management’ approach.

## Ethics Statement

The study was conducted in adherence to ethical guidelines. The study has been approved by the University of Texas Southwestern Institutional Review Board (study number: STU062015‐005). Clinical Trial registration: ENDPANIC, NCT02804438. Registered 1 July 2015.

## Consent

Study data were obtained from chart extracts after patient discharge and do not contain identifiable patient data. The University of Texas Southwestern Institutional Review Board determined that subject consent was not required.

## Conflicts of Interest

Dr Li received funding from Affiliated Hospital of Zunyi Medical University as a visiting professor. Dr Olson declares that he is the editor for the Journal of Neuroscience Nursing. All other authors report no conflicts of interest.

## Data Availability

The data that support the findings of this study are available on request from the corresponding author. The data are not publicly available due to privacy or ethical restrictions.
